# Supplementary material for: LRIG proteins regulate lipid metabolism via BMP signaling and affect the risk of type 2 diabetes
Source: Commun Biol. 2021 Jan 19;4:90. doi: 10.1038/s42003-020-01613-w (PMC7815736; doi:10.1038/s42003-020-01613-w)
Supplement: Supplementary file 1 — Supplementary Information [file 42003_2020_1613_MOESM1_ESM.pdf]

SUPPLEMENTARY INFORMATION

LRIG proteins regulate lipid metabolism via BMP signaling and affect the risk of type 2 diabetes

Carl Herdenberg[a], Pascal M. Mutie[b], Ola Billing[c], Ahmad Abdullah[a], Rona J. Strawbridge[d,e,f], Ingrid Dahlman[g], Simon Tuck[h], Camilla Holmlund[a], Peter Arner[g], Roger Henriksson[a], Paul W. Franks[b], Håkan Hedman[a,*]

[a]Department of Radiation Sciences, Umeå University, SE-90187 Umeå, Sweden. [b]Genetic and Molecular Epidemiology Unit, Department of Clinical Sciences, Lund University, Skane University Hospital, Malmo, Sweden. [c]Department of Surgical and Perioperative Sciences, Surgery, Umeå University, SE-90187 Umeå, Sweden. [d]Institute of Health and Wellbeing, University of Glasgow, Glasgow, UK. [e]Department of Medicine Solna, Karolinska Institutet, Stockholm, Sweden. [f]Health Data Research UK, University of Glasgow, Glasgow, UK. [g]Department of Medicine Huddinge, Karolinska Institutet, Stockholm, Sweden. [h]Umeå Center for Molecular Medicine, Umeå University, SE-90187 Umeå, Sweden.

*Corresponding author; e-mail: hakan.hedman@umu.se

Supplementary Figure1

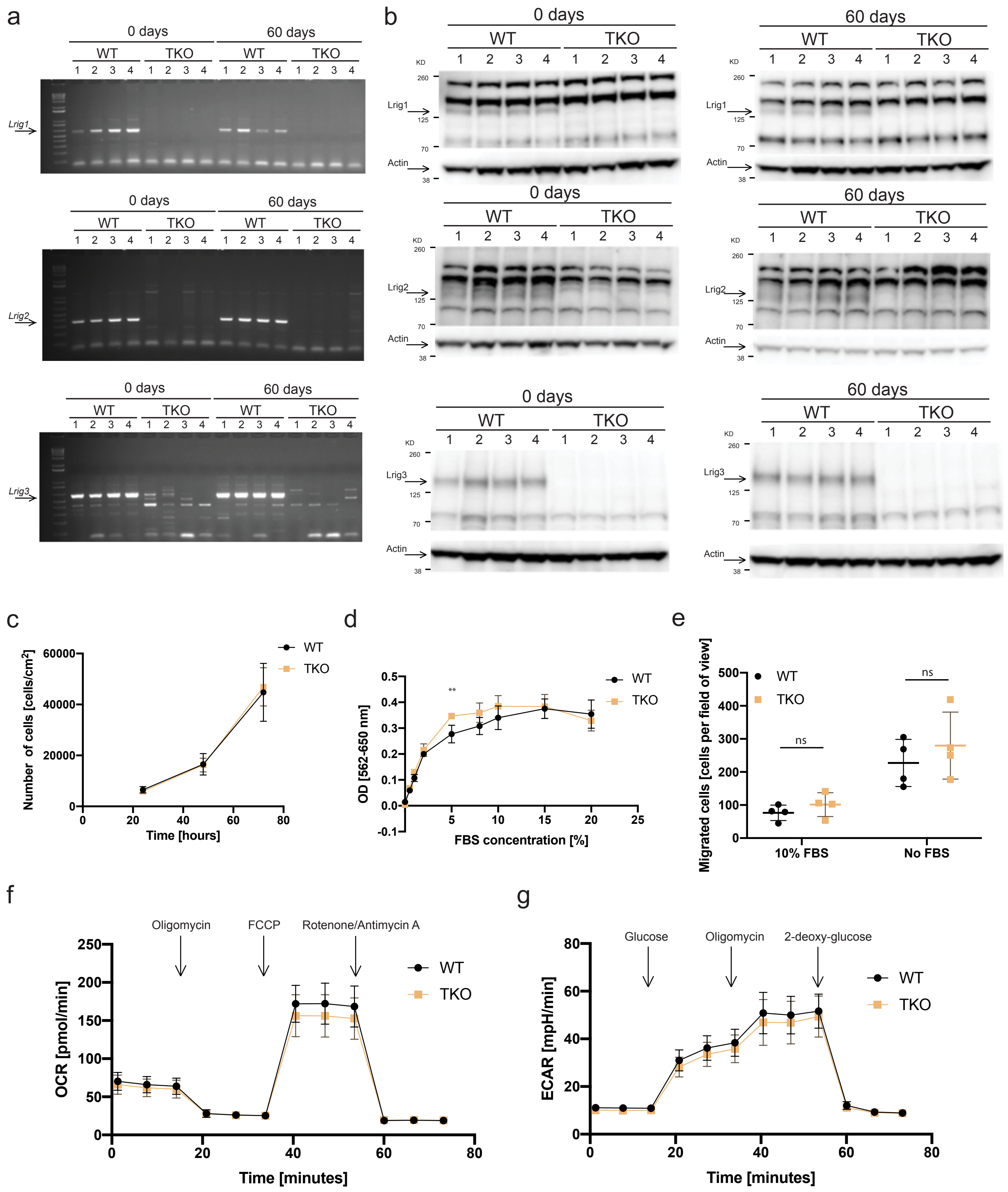

Supplementary Figure 1: Characterization of wild-type (WT) and *Lrig*-null (TKO) mouse embryonic fibroblasts (MEFs). MEFs with floxed *Lrig1*, *Lrig2*, and *Lrig3* alleles were transiently transduced with Cre recombinase-expressing adenovirus or transduction control adenovirus on four different occasions, creating four MEF line pairs, each consisting of a wild-type (WT1-4) and an *Lrig*-null (TKO1-4) cell line. **a** Images showing agarose gels for PCR genotyping of *Lrig1*, *Lrig2*, and *Lrig3* gene loci. DNA was prepared at day 0 and after 60 days of cell cultivation. Arrows indicate the expected size of the respective wild-type *Lrig* band. **b** Lrig Western blot analyses. Protein samples were prepared at the indicated times of cultivation (0 or 60 days), separated by SDS page followed by Western blotting using antibodies specific for Lrig1, Lrig2, Lrig3, and actin. Specific bands are indicated with arrows. **c** Graph showing cell proliferation of wild-type and *Lrig*-null MEFs, measured by the number of cells per cm^2 at 24, 48 and 72 hours after plating. **d** Cell proliferation assay showing apparent cell viability at different FBS concentrations. Wild-type and *Lrig*-null MEFs were seeded at different FBS concentrations. Forty-eight hours after seeding, the relative number of cells was quantified using an MTT assay. Shown are the OD values from the MTT assay. **e** Cell migration assay with wild-type and *Lrig*-null MEFs. Cells were seeded in the upper compartments of transwell chambers with the lower compartment containing standard culture medium with or without 10% FBS. Twenty-four hours after the cell seeding, the transmigrated cells were fixated, stained with crystal violet, and manually counted under a microscope. **f** Aerobic metabolism: oxygen consumption rates (OCR) were analyzed for wild-type and *Lrig*-null MEFs on a Seahorse XF instrument. The indicated reagents were added at the indicated times. **g** Anaerobic metabolism: extracellular acidification rates (ECAR) were analyzed for wild-type and *Lrig*-null MEFs on a Seahorse XF instrument. The indicated reagents were added at the indicated times. The plotted values in c-g represent means from four biological replicates, each with three experimental repeats. The error bars represent the standard deviations of means from four biological replicates. ^{ns} $P>0.05$, ** $P<0.01$ (Student's t-test).

Supplementary Figure 2

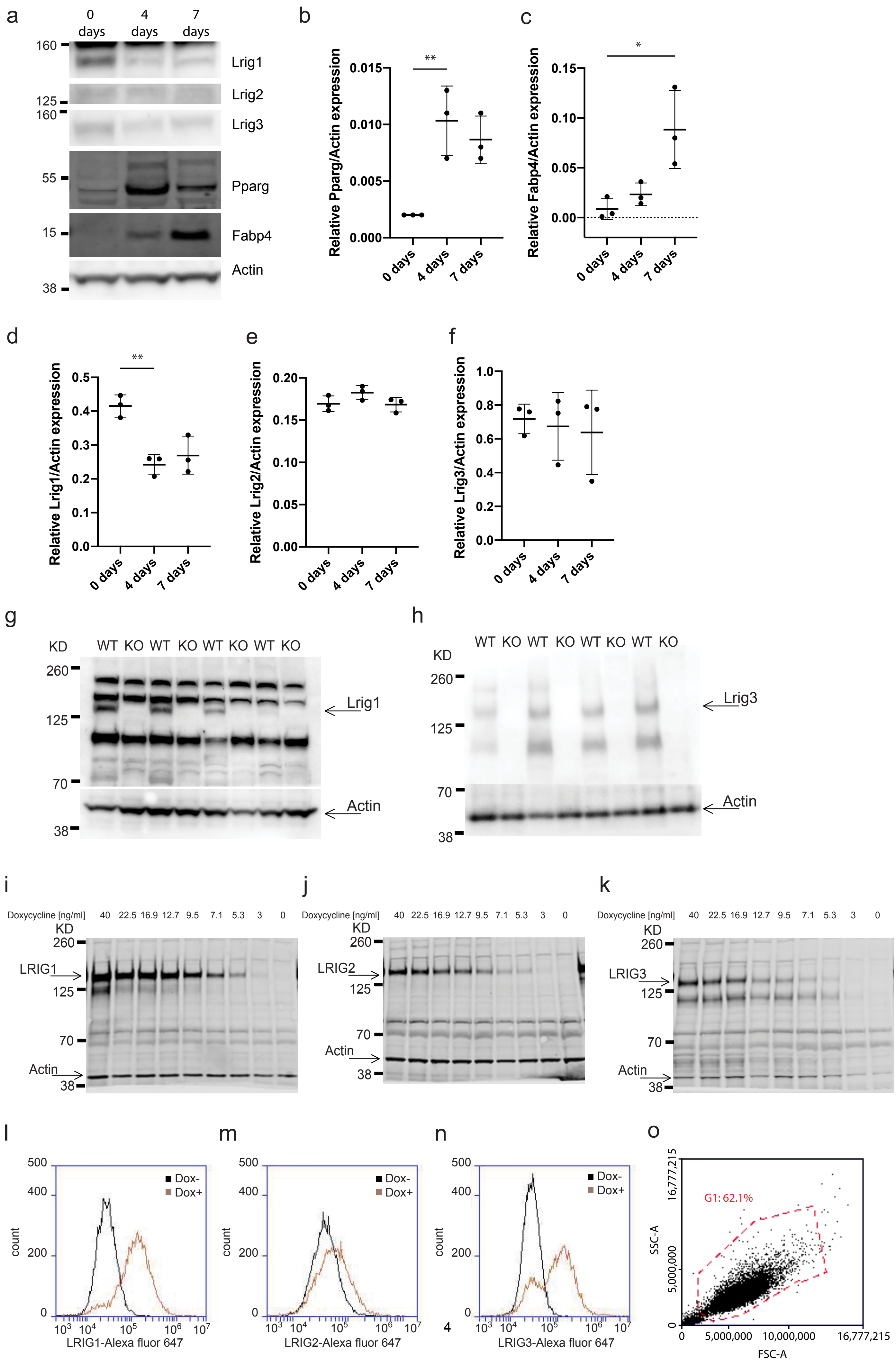

Supplementary Figure 2: LRIG protein expression analyses of single *Lrig*-knockout and *LRIG*-inducible MEFs. a Representative Western blots for Lrig1, Lrig2, Lrig3, Pparg, Fabp4, and actin, which was used as loading control, at 0, 4, and 7 days after the adipogenic induction of wild-type MEFs. **b-f** Quantifications of the Western blots of the respective protein at 0, 4, and 7 days after adipogenic induction. Shown are the results of three independent experiments. **b** Relative expression of Pparg/actin. **c** Relative expression of Fabp4/actin. **d** Relative expression of Lrig1/actin. **e** Relative expression of Lrig2/actin duction. **f** Relative expression of Lrig3/actin. **g** Western blot analysis showing the protein expression of Lrig1 and actin in wild-type (WT) and *Lrig1*-null (KO) MEF clones. **h** Western blot analysis showing the protein expression of Lrig3 and actin in wild-type (WT) and *Lrig3*-null (KO) MEF clones. **i-k** Western blots showing LRIG protein expression in LRIG-inducible MEF lines that had been treated with different concentrations of doxycycline. Shown are blots for LRIG1-inducible cells (i), LRIG2-inducible cells (j), and LRIG3-inducible cells (k). LRIG proteins were detected with an anti-FLAG antibody recognizing a FLAG epitope present on the induced LRIG proteins. Actin was used as the protein loading control. **l-o** Flow cytometry analyses on *Lrig*-null cell lines with inducible *LRIG1*, *LRIG2*, or *LRIG3* FLAG-tagged genes. LRIG protein expression was induced by treatment with 1 µg/ml doxycycline for 24 hours, followed by anti-FLAG antibody staining and flow cytometry analysis. The overlay histograms show LRIG1-inducible cells (l), LRIG2-inducible cells (m), and LRIG3-inducible cells (n) with or without induction by doxycycline treatment. The cells were gated according to their side scatter (SSC) and forward scatter (FSC); an example of the gating strategy is shown in (o). Ten-thousand G1-gated events were acquired for each histogram

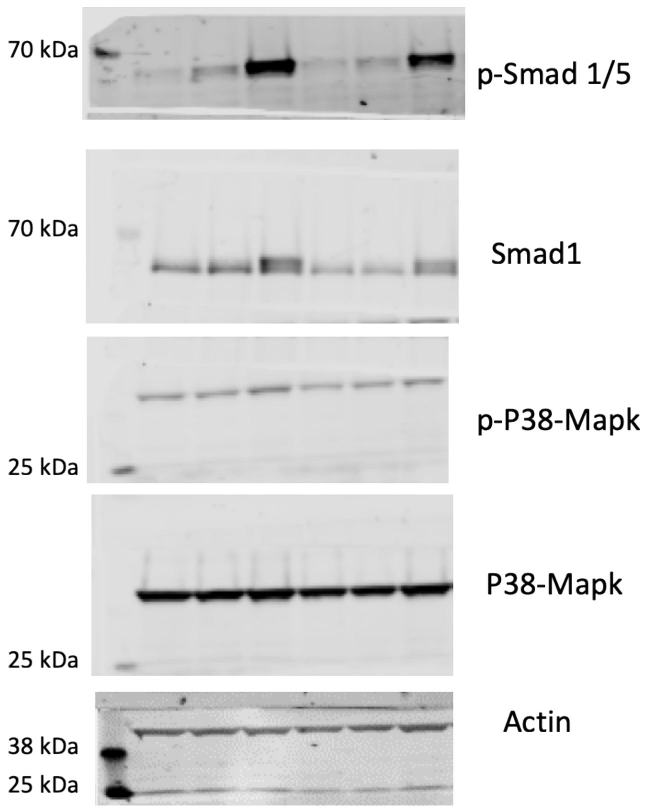

Supplementary Figure 3: Uncropped scans of Western blots in Fig. 2g.

Supplementary Figure 4

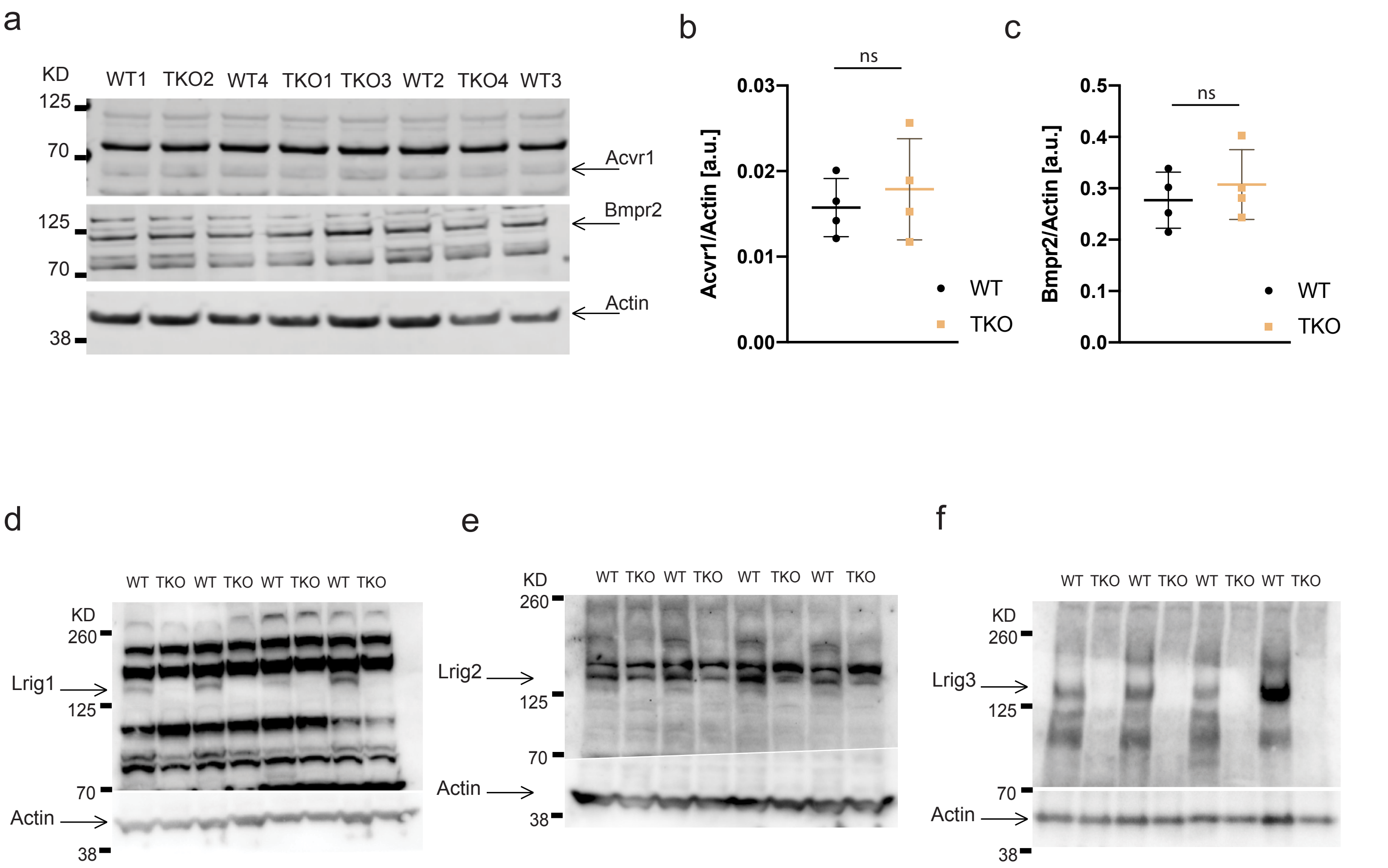

Supplementary Figure 4: BMP receptor levels of wild-type and Lrig-null MEF lines and genotyping of MAPK reporter cell lines via Western blotting. a-c Western blot analysis of Bmpr2, Acvr1, and actin in wild-type (WT) and Lrig-null (TKO) MEF lines. a Representative Western blots for Acvr1, Bmpr2, and actin, which was used as loading control. b Quantifications of the relative expression of Acvr1 in wild-type and Lrig-null MEFs. Shown are the results of three experimental repeats using four biological replicates of each genotype. c Quantifications of the relative Bmpr2 expression in wild-type and Lrig-null MEFs. Shown are the results of three experimental repeats using four biological replicates of each genotype. d-f An MEF line with floxed Lrig alleles was stably transduced with the MAPK luciferase reporter construct ELK1/SRF-luc. Thereafter, the cells were transiently transduced with Cre recombinase-expressing adenovirus or transduction control adenovirus on four different occasions, generating four wild-type MAPK reporter MEF lines (WT) and four Lrig-null MAPK reporter MEF lines (TKO). The Western blots show Lrig1 and actin (d), Lrig2 and actin (e), and Lrig3 and actin (f) protein bands for the four wild-type (WT) and the four Lrig-null (KO) MAPK reporter MEF lines. The expected sizes of the respective LRIG proteins and actin are indicated with arrows.

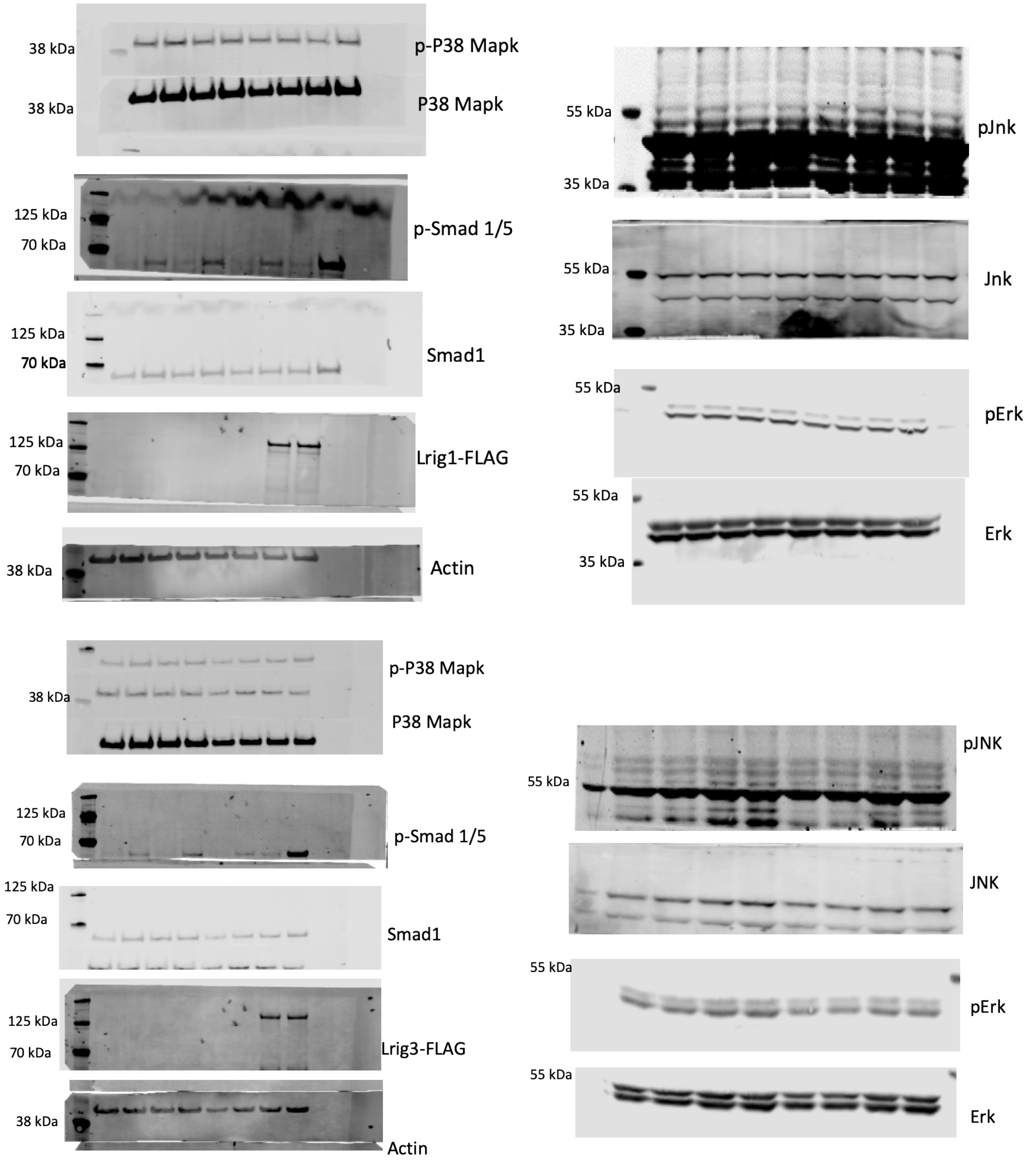

Supplementary Figure 5: Uncropped scans of Western blots in Fig. 3e, f.

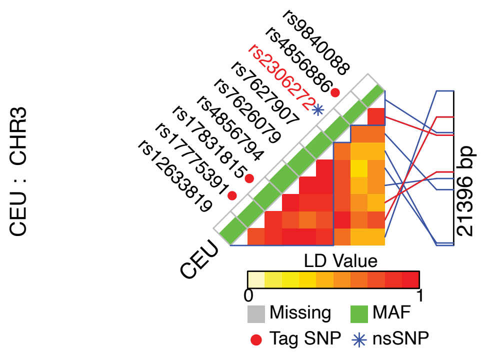

Supplementary Figure 6: LRIG1 tag SNPs identified using online tool, SNPinfo. To the left is information on population and chromosome name and to the right is the length of the chromosome within which the SNPs are contained. The green bars represent the minor allele frequency of each SNP in the selected population, CEU in this case. Tag SNPs are indicated by red dots, while a blue asterisk indicates a non-synonymous SNP.

Supplementary Tables

Supplementary Table 1. Fraction of *Lrig3* knockout MEFs in the wild-type (WT1-4) and *Lrig*-null (TKO1-4) MEF lines.

	0 days	60 days
	Ratio (*Lrig3* exon1/reference exon)[a,b] (mean ±standard deviation)	Ratio (*Lrig3* exon1/reference exon)[a,b] (mean ± standard deviation)
WT1	0.975 ± 0.035	0.960 ± 0.015
WT2	0.944 ± 0.020	1.013 ± 0.045
WT3	0.981 ± 0.029	1.469 ± 0.819
WT4	1.105 ± 0.118	1.040 ± 0.025
TKO1	0.001 ± 0.000	0.000 ± 0.000
TKO2	0.004 ± 0.002	0.003 ± 0.002
TKO3	0.000 ± 0.000	0.001 ± 0.000
TKO4	0.000 ± 0.000	0.000 ± 0.000

[a] The proportion (ratio) of *Lrig3* knockout MEFs was determined by using a duplex ddPCR for *Lrig3* exon 1together with a reference locus.
[b] Shown are the means and standard deviation of three technical replicates.

Supplementary Table 2. Forward scatter and side scatter mean values from wild-type (WT1-4) and *Lrig*-null (TKO1-4) MEF lines.

	Forward scatter[a] (mean ± standard deviation)	Side scatter[a] (mean ± standard deviation)
WT1	7269 ± 111	938 ± 101
WT2	7680 ± 159	991 ± 45
WT3	6769 ± 156	907 ± 88
WT4	7388 ± 233	1008 ± 71
TKO1	7342 ± 36	934 ± 65
TKO2	7594 ± 131	985 ± 94
TKO3	6153 ± 290	768 ± 87
TKO4	7476 ± 96	1040 ± 117

[a] Shown are the means and standard deviation of three technical replicates.

Supplementary Table 3. Participants' characteristics, UK Biobank.

Characteristic	Men	Women
Age (mean, SD), yrs	57.2 (8.1)	56.7 (7.9)
BMI (mean, SD), kg/m^2	27.6 (3.8)	26.6 (4.4)
Diabetes (N, %)		
No	172741 (54.7%)	208199 (45.3%)
Yes	6546 (36.6%)	11324 (63.4%)
Cholesterol (mean, SD), mmol/L	5.5 (1.1)	5.9 (1.1)
Triglycerides (mean, SD), mmol/L	2.0 (1.1)	1.6 (0.9)
Liver fat % (mean, SD)	4.4 (4.6)	3.3 (4.2)

Supplementary Table 4. Genome-wide association significant *LRIG1* SNPs, UK Biobank.

RS_id	Position	Allele1	Allele2	MAF	Minor Allele	Info score
rs7627907	66426912	A	G	0.340631	G	0.9981
rs7626079	66427259	C	T	0.345515	T	1
rs2306272	66434643	T	C	0.287853	C	1
rs4856794	66436182	T	G	0.349079	G	0.996736
rs17831815	66437086	T	C	0.346928	C	0.995861
rs4856886	66442180	T	G	0.196361	G	0.982886
rs9840088	66442545	A	C	0.204582	C	0.983441
rs17775391	66444697	A	C	0.286741	C	0.992446
rs12633819	66448307	A	G	0.342479	G	0.989515
Bold = tag SNPs						

Supplementary Table 5. Association between *LRIG1* tag SNPs and BMI† in the UK Biobank.

SNP_ref allele	ß estimate	Std. error	95% CI	P value
rs7627907_G	0.036	0.0098	0.017, 0.055	2.4×10^{-4}
rs7626079_T	0.04	0.0098	0.021, 0.059	4.7×10^{-5}
rs2306272_C	0.04	0.01	0.02, 0.061	8.2×10^{-5}
rs4856794_G	0.038	0.0098	0.018, 0.057	1.2×10^{-4}
rs17831815_C[a]	0.038	0.0098	0.019, 0.057	1×10^{-4}
rs4856886_G[a]	0.052	0.012	0.029, 0.075	1.2×10^{-5}
rs9840088_C	0.052	0.012	0.029, 0.075	7.6×10^{-6}
rs17775391_C[a]	0.039	0.01	0.019, 0.059	1.3×10^{-4}
rs12633819_G	0.036	0.0098	0.017, 0.055	2.5×10^{-4}

†Untransformed residuals used as outcome
[a] Tag SNPs.

Supplementary Table 6. Association between *LRIG1* SNPs and the risk of type 2 diabetes in the UK Biobank.

	Not adjusted for BMI		Adjusted for BMI[†]	
SNP_ref allele	OR (95% CI)	P value	OR (95% CI)	P value
rs7627907_G	0.967 (0.945, 0.989)	3.67×10^{-3}	0.961 (0.939, 0.983)	7.23×10^{-4}
rs7626079_T	0.967 (0.945, 0.989)	3.68×10^{-3}	0.96 (0.938, 0.983)	5.55×10^{-4}
rs2306272_C	0.971 (0.948, 0.994)	1.38×10^{-2}	0.964 (0.941, 0.987)	2.85×10^{-3}
rs4856794_G	0.968 (0.947, 0.99)	5.22×10^{-3}	0.962 (0.94, 0.985)	1.03×10^{-3}
rs17831815_C[a]	0.968 (0.946, 0.99)	4.89×10^{-3}	0.962 (0.94, 0.984)	9.57×10^{-4}
rs4856886_G[a]	0.964 (0.938, 0.991)	9.09×10^{-3}	0.954 (0.928, 0.981)	9.33×10^{-4}
rs9840088_C	0.964 (0.938, 0.99)	8.02×10^{-3}	0.954 (0.928, 0.981)	8.53×10^{-4}
rs17775391_C[a]	0.971 (0.948, 0.994)	1.38×10^{-2}	0.964 (0.941, 0.987)	2.88×10^{-3}
rs12633819_G	0.965 (0.943, 0.987)	2.25×10^{-3}	0.959 (0.937, 0.982)	4.38×10^{-4}

[†] Using untransformed BMI residuals
[a] Tag SNPs.

Supplementary Table 7. Association between *LRIG1* SNPs and triglyceride† levels in the UK Biobank.

SNP	ß estimate	Std. error	95% CI	P value
rs7627907_G	-0.0034	0.0024	-0.0082, 0.0013	0.15
rs7626079_T	-0.003	0.0024	-0.0078, 0.0017	0.21
rs2306272_C	-0.0034	0.0025	-0.0083, 0.0016	0.19
rs4856794_G	-0.0036	0.0024	-0.0083, 0.0011	0.13
rs17831815_C[a]	-0.0037	0.0024	-0.0085, 0.001	0.12
rs4856886_G[a]	-0.0073	0.0029	-0.013, -0.0016	0.012
rs9840088_C	-0.0063	0.0029	-0.012, -0.00069	0.028
rs17775391_C[a]	-0.0044	0.0025	-0.0093, 0.00061	0.086
rs12633819_G	-0.0031	0.0024	-0.0078, 0.0017	0.21

†Inverse normal transformed residuals used as the outcome.
[a] Tag SNPs.

Supplementary Table 8. Associations between *LRIG1* SNPs and liver fat percentage† in the UK Biobank (n = 3,192).

SNP	ß estimate	Std. error	95% CI	P value
rs7627907_G	0.015	0.024	-0.032, 0.062	0.53
rs7626079_T	0.026	0.024	-0.022, 0.073	0.29
rs2306272_C	-0.019	0.025	-0.068, 0.03	0.45
rs4856794_G	0.011	0.024	-0.037, 0.058	0.66
rs17831815_C[a]	0.0072	0.024	-0.040, 0.054	0.76
rs4856886_G[a]	-0.012	0.029	-0.068, 0.045	0.69
rs9840088_C	-0.0074	0.028	-0.063, 0.048	0.8
rs17775391_C[a]	-0.011	0.025	-0.061, 0.038	0.65
rs12633819_G	0.018	0.024	-0.029, 0.066	0.46

†Inverse normal transformed liver fat % residuals used as the outcome.
[a] Tag SNPs.

Supplementary Table 9. Associations between *LRIG1* SNPs and adipose morphology.

SNP	CHR	POS	REF	ALT	TEST	OBS_CT	BETA	SE	L95	U95	T_STAT	P	Ref freq
rs4856886	3	66442180	G	T	ADD	894	20.2	9.8	39.4	1.1	2.07	0.039	0.19
rs9840088	3	66442545	C	A	ADD	894	23.7	9.7	42.7	4.7	2.45	0.015	0.19

Supplementary Table 10. Antibodies used in the study.

Antigen	Host	Company	Catalog no.	Lot no.	Application[a]	Dilution
Lrig1	Rabbit	N/A: generated in-house	mLrig1-255	2532U	WB	1:500
Lrig2	Rabbit	Agrisera	AS142788	2534U	WB	1:350
Lrig3	Rabbit	Agrisera	AS142789	2536U	WB	1:500
PPARγ	Rabbit	Cell Signaling Technology	2435	6	WB	1:1,000
FABP4	Rabbit	Cell Signaling Technology	2120	3	WB	1:1,000
Actin	Mouse	Abcam	ab3280	gr297708-1	WB	1:5,000
Actin	Rabbit	Sigma-Aldrich	A2066	057k4803	WB	1:7,000
Smad1	Rabbit	Cell Signaling Technology	6944S	5	WB	1:1,000
pSmad1/5	Rabbit	Cell Signaling Technology	9516	9	WB, ICC	1:800
p38	Rabbit	Cell Signaling Technology	9212S	26	WB	1:1,000
phospho-p38	Rabbit	Cell Signaling Technology	9211S	24	WB	1:1,000
SAPK/JNK	Rabbit	Cell Signaling Technology	9252s	17	WB	1:1,000
Phospho-SAPK/JNK	Mouse	Cell Signaling Technology	9255s	33	WB	1:2,000
p44/42 MAPK (Erk1/2)	Rabbit	Cell Signaling Technology	4695s	28	WB	1:1,000
Phospho-p44/42 MAPK (Erk1/2)	Rabbit	Cell Signaling Technology	4370s	24	WB	1:2,000
FLAG M2	Mouse	Sigma-Aldrich	F3165	SLBT6752	WB, FACS, ICC	1:5,000, 1:1,000, 1:5,000

ACVR1	Rabbit	Novus biologicals	NBP1-33500	40142	WB	1:500
Bmpr2	Mouse	Fisher Scientific	3F6F8	td269789	WB	1:500
Mouse IgG HRP		GE Healthcare	na9310v	10370044	WB	1:20,000
Rabbit IgG HRP		GE Healthcare	na934vs	9833234	WB	1:20,000
Mouse IgG IRDye 800CW	Goat	LI-COR Biosciences	926-32210	C31021-01	WB	1:15,000
Rabbit IgG IRDye 680RD	Goat	LI-COR Biosciences	925-68071	C60329-11	WB	1:15,000
Alexa fluor anti-rabbit 647	Goat	Invitrogen	A-21245	2051068	FACS, ICC	1:1,000
Alexa fluor anti-mouse 488	Donkey	Invitrogen	A-21202	1305303	ICC	1:1,000
pSmad3	Rabbit	Abcam	ab52903	gr3194559-10	ICC	1:1,000

[a]WB, Western blotting; ICC, immunocytochemistry; FACS, flow cytometry
