# Supplementary material for: LRIG proteins regulate lipid metabolism via BMP signaling and affect the risk of type 2 diabetes
Source: Commun Biol. 2021 Jan 19;4:90. doi: 10.1038/s42003-020-01613-w (PMC7815736; doi:10.1038/s42003-020-01613-w)
Supplement: Supplementary file 2 — Description of Additional Supplementary Files [file 42003_2020_1613_MOESM2_ESM.pdf]

Description of Additional Supplementary Files

File Name: Supplementary Data 1
Description: All source data underlying the graphs in the main figures.
