# Supplementary material for: LRIG proteins regulate lipid metabolism via BMP signaling and affect the risk of type 2 diabetes
Source: Commun Biol. 2021 Jan 19;4:90. doi: 10.1038/s42003-020-01613-w (PMC7815736; doi:10.1038/s42003-020-01613-w)
Supplement: Supplementary file 4 — Reporting Summary [file 42003_2020_1613_MOESM4_ESM.pdf]

nature research

Corresponding author(s): Håkan Hedman

Last updated by author(s): Nov 2, 2020

Reporting Summary

Nature Research wishes to improve the reproducibility of the work that we publish. This form provides structure for consistency and transparency in reporting. For further information on Nature Research policies, see Authors & Referees and the Editorial Policy Checklist .

Statistics

For all statistical analyses, confirm that the following items are present in the figure legend, table legend, main text, or Methods section.

n/a	Confirmed	
☐	☒	The exact sample size (n) for each experimental group/condition, given as a discrete number and unit of measurement
☐	☒	A statement on whether measurements were taken from distinct samples or whether the same sample was measured repeatedly
☐	☒	The statistical test(s) used AND whether they are one- or two-sided<br>*Only common tests should be described solely by name; describe more complex techniques in the Methods section.*
☐	☒	A description of all covariates tested
☐	☒	A description of any assumptions or corrections, such as tests of normality and adjustment for multiple comparisons
☐	☒	A full description of the statistical parameters including central tendency (e.g. means) or other basic estimates (e.g. regression coefficient) AND variation (e.g. standard deviation) or associated estimates of uncertainty (e.g. confidence intervals)
☐	☒	For null hypothesis testing, the test statistic (e.g. F, t, r) with confidence intervals, effect sizes, degrees of freedom and P value noted<br>*Give P values as exact values whenever suitable.*
☒	☐	For Bayesian analysis, information on the choice of priors and Markov chain Monte Carlo settings
☒	☐	For hierarchical and complex designs, identification of the appropriate level for tests and full reporting of outcomes
☐	☒	Estimates of effect sizes (e.g. Cohen's d, Pearson's r), indicating how they were calculated

Our web collection on statistics for biologists contains articles on many of the points above.

Software and code

Policy information about availability of computer code

Data collection	QX200 droplet reader (Bio-Rad Laboratories AB), Odyssey CLx imaging system (LI-COR Biosciences GmbH) ,ChemiDoc Touch Imaging System (Bio-Rad Laboratories AB), BD Accuri C6 instrument (BD Biosciences),Synergy2 microplate reader (BioTek Instruments SAS, Colmar Cedex, France), Seahorse XFe cell analyzer (Agilent Technologies, Inc., Santa Clara, CA, USA), Spectramax i3x plate reader (Molecular Devices, San Jose, CA, USA), CFX96 system C1000 thermal cycler (Bio-Rad Laboratories AB), Glomax 96 microplate luminometer (Promega Biotech AB),Trophos Plate Runner HD (Trophos/Dioscure, Marseille, France),Ion Technology sequencer Ion Proton (Thermo Fisher Scientific)
Data analysis	QuantaSoft software (Bio-Rad Laboratories AB, version 1.7.4.0917), Softmax Pro 7 software (Molecular Devices),Tina analysis package (Trophos/Dioscure), STAR, bowtie2, HTSeq, Fiji software, snptag, an online tool of SNPinfo, GENIAL study Samples were genotyped using the UK Biobank Axiom Array r3 platform and called using the Axiom analysis suite. PLINK (http://pngu.mgh.harvard.edu/purcell/plink/)

For manuscripts utilizing custom algorithms or software that are central to the research but not yet described in published literature, software must be made available to editors/reviewers. We strongly encourage code deposition in a community repository (e.g. GitHub). See the Nature Research guidelines for submitting code & software for further information.

Data

Policy information about availability of data

All manuscripts must include a data availability statement. This statement should provide the following information, where applicable:

- Accession codes, unique identifiers, or web links for publicly available datasets
- A list of figures that have associated raw data
- A description of any restrictions on data availability

The datasets generated during and/or analysed during the current study are available from the corresponding author on reasonable request.

Field-specific reporting

Please select the one below that is the best fit for your research. If you are not sure, read the appropriate sections before making your selection.

[x] Life sciences [ ] Behavioural & social sciences [ ] Ecological, evolutionary & environmental sciences

For a reference copy of the document with all sections, see nature.com/documents/nr-reporting-summary-flat.pdf

Life sciences study design

All studies must disclose on these points even when the disclosure is negative.

Sample size	Most cell experiments were done using four biological replicates using three experimental repeats.
Data exclusions	For cell and animal experiments: no biological replicates, or experimental replicates were excluded in this study.<br>In the UK Biobank study, we excluded participants who did not have BMI measurements or of any of the other outcome variables of interest (done at the stage of that particular analysis) and those who had ambiguous information on sex (discordance between self-reported and genetically encoded sex).<br>In the GENIAL study samples were excluded for cryptic relatedness, ambiguous sex, or low call rate (<95%). Population outliers were excluded by visual inspection of PCs1-4. SNPs were excluded for low call rate (<95%), failing Hardy-Weinberg equilibrium (p <5x10-6) or low MAF (<1%). After quality control, imputation was performed to the haplotype reference consortium panel and, when variants were not available, to the 1000G phase3 reference panel [Loh et al., 2016]. Postimputation quality control excluded related individuals (one of each pair of 1st or 2nd degree relatives).
Replication	The large majority of experiments were performed using four biological replicates, where each biological replicate was analyzed through three independent experimental repeats.
Randomization	Plate and well order in cell experiments were observed and showed no effect on results.
Blinding	In as many experiments as possible the investigators were blinded until after data analysis. In some cases this was not possible due to practical reasons.

Reporting for specific materials, systems and methods

We require information from authors about some types of materials, experimental systems and methods used in many studies. Here, indicate whether each material, system or method listed is relevant to your study. If you are not sure if a list item applies to your research, read the appropriate section before selecting a response.

Materials & experimental systems

n/a	Involved in the study	
[ ]	[x]	Antibodies
[ ]	[x]	Eukaryotic cell lines
[x]	[ ]	Palaeontology
[ ]	[x]	Animals and other organisms
[ ]	[x]	Human research participants
[x]	[ ]	Clinical data

Methods

n/a	Involved in the study	
[x]	[ ]	ChIP-seq
[ ]	[x]	Flow cytometry
[x]	[ ]	MRI-based neuroimaging

Antibodies

Antibodies used	All antibodies used are listed in Table S10.
Validation	Ppary: Commercial rabbit monoclonal antibody from Cell Signaling Technology. It recognized bands of the expected molecular weights, 53 and 57 kDa (Supplementary Figure 2a). No further validation was performed.; Fabp4: Commercial rabbit polyclonal antibody from Cell Signaling Technology. It recognized a band of the expected molecular weight, 15 kDa (Supplementary Figure 2a). No further validation was performed.; FLAG M2: Internally validated through immunofluorescence of cells and immunoblotting of cell lysates from samples that had been induced, or not induced, or transfected with different amounts of expression plasmids, to express FLAG epitope-tagged fusion proteins, e.g., Figures 3e,f and 4, and Supplementary Figure 2i-k. Bmpr2: Commercial mouse monoclonal antibody from Thermo Fisher. It recognized a band of the expected molecular weight, 115 kDa (Supplementary Figure 3a). No further validation was performed.; Lrig1: Internally validated through immunoblotting of tissues (not shown) and cell lysates from wild-type and Lrig1-null samples, e.g., Supplementary Figure 1b. Lrig2: Internally validated through immunoblotting of tissues (not shown) and cell lysates from wild-type and Lrig2-null samples, e.g., Supplementary Figure 1b. Lrig3: Internally validated through immunoblotting of tissues (not shown) and cell lysates from wildtype and Lrig3-null samples, e.g., Supplementary Figure 1b. Actin: Mouse monoclonal antibody from Abcam. It recognized a prominent band of the expected molecular weight, 42 kDa. No further validation was performed. Actin: Affinity-purified polyclonal antibody from Sigma-Aldrich. It recognized a prominent band of the expected molecular weight, 42 kDa. No further

	validation was performed. pSmad1/5: Internally validated through cell immunofluorescence or immunoblotting of cells or cell lysates from cells treated, or not treated, with BMP4, 6, or 9. Figures 2b-h, 3a-f,n,o, 4. p38: Rabbit monoclonal antibody from Cell Signaling. Validated by the supplier through downregulation of the expected 40 kDa band through siRNA. phospho-p38: Internally validated through immunoblotting of cell lysates from cells treated, or not treated, with BMP4. The band also showed the same molecular weight as p38. Figure 3e,f. SAPK/JNK: Rabbit polyclonal antibody from Cell Signaling. Validated by the supplier through immunoblotting and detection of the expected 46 and 54 kDa bands. phospho-SAPK/JNK: Internally validated through immunoblotting of cell lysates from cells treated, or not treated, with anisomycin. The bands also showed the same molecular weights as SAPK/JNK. p44/42 MAPK (Erk1/2): Rabbit monoclonal antibody from Cell Signaling. Validated by the supplier through downregulation of the expected 44 and 42 kDa bands through siRNA. phospho-p44/42 MAPK (Erk1/2): Rabbit monoclonal antibody from Cell Signaling. Internally validated through immunoblotting of cell lysates from cells treated, or not treated, with PDGF-BB. The band also showed the same molecular weight as p44/42 MAPK. Smad1: Rabbit monoclonal antibody from Cell Signaling. Recognized a band of the same molecular weight as pSmad1/5. No further validation was performed. Acvr1: Commercial antibody from Novus. It recognized a band of the expected molecular weight, 57 kDa (Supplementary Figure 3a). No further validation was performed.; pSmad3: Internally validated through the demonstration of a dose-response of the specific immunoblot band vis-a-vis the amount of TGF-beta1 that was used to stimulate the cells (not shown).

Eukaryotic cell lines

Policy information about cell lines

Cell line source(s)	Cell line source(s): All mouse embryonic fibroblast (MEF) lines were generated in-house according to the 3T3 protocol. HEK293T (293T) was obtained from ATCC via GeneScript. A375 was obtained from ATCC.
Authentication	In all experiments, the MEF Lrig genotypes were confirmed through immunoblotting; i.e., wildtype or Lrig-null, with or without inducible human LRIG alleles. The HEK293T (293T) cells were obtained from GeneScript in March, 2017. Thereafter the 293T genotype was not further confirmed, however, the LRIG1 genotypes were assessed in all experiments through immunoblotting; i.e., it was confirmed that the cells were LRIG1-null with an inducible LRIG1 allele. A375 was authenticated in April, 2018 through STR profiling that was performed by ATCC's cell line authentication service.
Mycoplasma contamination	All cell lines were routinely tested for mycoplasma contamination and no cell lines tested positive.
Commonly misidentified lines (See ICLAC register)	NA

Animals and other organisms

Policy information about studies involving animals; ARRIVE guidelines recommended for reporting animal research

Laboratory animals	Mouse (Mus musculus) strains B6.129-Lrig1tm1Hhed, B6.129-Lrig2tm1Hhed, B6.129-Lrig3tm1Hhed, B6.129-Lrig1tm1.1Hhed, and B6.129-Lrig3tm1.1Hhed on a C57BL/6J genetic background. C. elegans strains N2 Bristol (wild-type), CB907 (dpy-5(e907)), LT577 (sma-10(wk88)), LT578 (sma-10(wk89)), DR63 (daf-4(m63)), LT186 (sma-6(wk7)), VB942 (sma-3(wk30))
Wild animals	NA
Field-collected samples	NA
Ethics oversight	NA

Note that full information on the approval of the study protocol must also be provided in the manuscript.

Human research participants

Policy information about studies involving human research participants

Population characteristics	The UK Biobank is a large project with genotyped and well-phenotyped individuals comprising approximately 500,000 participants [Sudlow et al., 2015]. In this study, we excluded participants who did not have BMI measurements or of any of the other outcome variables of interest (done at the stage of that particular analysis) and those who had ambiguous information on sex (discordance between self-reported and genetically encoded sex). The final sample size was 398,810 participants of Caucasian ancestry.<br>The GENetIcs of Adipocyte Lipolysis (GENIAL) cohort included 273 men and 718 women. Fifty-seven percent of the participants were obese (defined as BMI >30kg/m2). All lived in the Stockholm County, Sweden and were at least second-generation Swedes. One hundred ninety-four participants had type 2 diabetes, hypertension, or dyslipidemia alone or in different combinations. None were treated with insulin, glitazones, or glucagon-like-peptide analogs.
Recruitment	Subjects in the GENIAL cohort were recruited by local advertisement to examine the regulation of fat cell function.
Ethics oversight	The UK Biobank study was conducted using publicly available data and therefore did not require a specific ethical approval. The GENIAL study was approved by the local committee on ethics

Note that full information on the approval of the study protocol must also be provided in the manuscript.

Flow Cytometry

Plots

Confirm that:

- [x] The axis labels state the marker and fluorochrome used (e.g. CD4-FITC).
- [x] The axis scales are clearly visible. Include numbers along axes only for bottom left plot of group (a 'group' is an analysis of identical markers).
- [x] All plots are contour plots with outliers or pseudocolor plots.
- [x] A numerical value for number of cells or percentage (with statistics) is provided.

Methodology

Sample preparation	Cells were dissociated using Accutase cell detachment solution and then washed in PBS containing 5% FBS. Cells were fixated in 4% phosphate-buffered formaldehyde, permeabilized using 0.2% saponin in PBS. The cells were labeled with primary antibodies for 30 minutes on ice, washed and then incubated with secondary antibodies for 30 minutes on ice.
Instrument	BD Accuri C6 instrument (BD Biosciences)
Software	Collected and analysed on BD FACS software
Cell population abundance	10,000 FSC/SSC gated events were collected.
Gating strategy	Forward scatter and side scatter was used to remove debris. No other gating strategy was used.

- [x] Tick this box to confirm that a figure exemplifying the gating strategy is provided in the Supplementary Information.
